# Does Menière’s Disease Occur in Horses?*Read before the Central Veterinary Society on May 7th, Vol. xxxii.

**Published:** 1891-07

**Authors:** George Fleming


### DOES MENIÈRE'S DISEASE OCCUR IN HORSES?*

BY GEORGE FLEMING, C.B., LL.D., F.R.C.V.S.

We know but little of the neurotic disorders of the domesticated animals, and are probably correct in inferring that they are very few in number, as compared with those affecting mankind. To account for this difference, we might invoke the extremely artificial life imposed upon our species, and especially upon dwellers in large towns; the highly-developed state of the nervous system, and the strain—mental particularly—to which it is subjected; the deteriorating effects of certain morbid conditions upon it—chiefly those due to Syphilis, Alcoholism, and Gout—as predisposing, if not immediately exciting, causes of various neuroses of a more or less serious character which find a prominent place in text-books on human pathology. Mental diseases veterinary surgeons have seldom indeed to contend with in animals, unless it be vicious temper, due to mismanagement, rarely to natural defect.

Therefore it is that the list of neurotic affections known to us is indeed small, and attracts but little attention. But these may be more numerous than we suspect or realize, for we labor under a

* Read before the Central Veterinary Society on May 7th, Vol. xxxii.

great disadvantage in several respects in dealing with animals, and more especially in their not being able to describe their feelings by articulate sounds, when suffering from pain or inconvenience; and also from their being generally under our observation for only very brief periods, during which it is not possible to note everything that might lead us to form anything like an accurate notion of what may be amiss—symptoms of a morbid condition not being always present in the same intensity or form, and our means of investigation being generally exceedingly limited and imperfect.

For these reasons it is that the vertiginous disorders of animals are badly defined, and in the horse more especially are usually ascribed to two or three causes—as indigestion, cerebral tumor, or deranged circulation in the nervous system. But the observation of two cases which occurred in my own experience, inclines me to the belief that animals may suffer from vertigo due to some derangement in the auditory apparatus, similar to what occurs in man in what is known as "Menière's Disease," or technically, "Labyrinthine Vertigo"—an affection characterized by sudden attacks of giddiness, which are preceded or accompanied by noises in one or both ears.

As described in medical text-books, the commencement of one of these attacks in a person is marked by a sudden buzzing or humming sound in one or both ears—usually loudest in one. This is soon followed by vertigo, which may be either objective or subjective; or the patient may experience a sensation of falling, generally in a forward direction, but sometimes backwards or to one side. The individual reels and clutches at neighboring objects, but rarely falls; though occasionally he may do so, or in severe cases may be thrown violently to the ground. There is some doubt as to whether there is ever loss of consciousness; if there is, it is only momentary, as the patient can always give an exact account of his sensations after the attack is over.

The attack usually lasts a few minutes, and passes off gradually, leaving the patient deaf and with noises in the ears. Towards the end of the seizure he is pale, with a cold, clammy skin, and often experiences nausea, or even vomits. Lateral nystagmus is sometimes present during the attack, and apparent movement of objects from side to side is complained of. Subsequent attacks are liable to come on at varying intervals, and are generally announced by an increase in the tinnitus or ear-noise, which is generally more or less present. Indeed, a shrieking or a whistling noise is a common precursor of an attack. The intervals between the seizures gradually become shorter, until the patient is reduced to a state of permanent vertigo, liable to exacerbations. The deafness gradually becomes more marked and the tinnitus less observable. In some cases a spontaneous cure is effected when the deafness becomes absolute, the vertigo then disappearing. It is stated that vertigo, tinnitus, and deafness, when combined in one person, generally indicate an affection of the auditory labyrinth, either from actual disease—inflammation or hæmorrhage—or from disease of the middle ear causing pressure on it; but it is also admitted that obstruction of the Eustachian tube or of the external auditory meatus, as by the accumulation of cerumen, may give rise to the same symptoms.

It must be noted, however, that some medical authorities do not regard Menière's disease as a severe form of Labyrinthine Vertigo, but rather as a distinct affection. According to this view, it is characterized by a sudden and severe attack, such as that just described, in a person who had no previous ear disease. The seizure may never recur, and the disturbance is nearly always confined to one ear; while the deafness is intense. The tinnitus and giddiness diminish or cease after the attack, and there is no evidence of disease of the middle or external ear. In Labyrinthine Vertigo, on the other hand, it is asserted that there is usually pre-existing ear disease and severe tinnitus, with more or less deafness; the attacks are less sudden and more frequent, and the giddiness is not so great, but it is more chronic. Fagge believed that in true Menière's disease, there is no affection of the labyrinth or cochlea of the ear, but some disturbance of the centres for hearing and equilibrium.*

Diseases of the ear are not common in our patients, for with the exception of the dog and cat, in which the external ear is frequently in a morbid state, we rarely have derangements in this organ brought to our notice. Yet it is on record that affections of the external ear will induce symptoms in the dog and cat, analogous to those described as indicating the existence of Menière's disease or Labyrinthine Vertigo in man. Mégnin was the first, I believe, to direct attention to the fact that acariasis of the external ear will often produce epileptiform fits in cats; and Nocard has noticed the same result from the same cause in dogs. My friend, Mr. A. J. Sewell, M.R.C.V.S., informs me that in his experience cats

* Fowler's Dictionary of Practical Medicine, p. 511.

very frequently suffer from the presence of a small parasite, about the size of an ordinary acarus, in their ears, and which sets up such "tremendous" irritation that the poor animal will suddenly shake and scratch its ears, and when walking will go as if intoxicated, often falling on its side. Mr. Sewell has found the same parasite in dogs' ears.

I could detect nothing of this kind in the ears of the horses whose symptoms I will now refer to, and which appeared to me to bear so close a resemblance to some of those that characterize Meniére's disease, that I have bean induced to bring the question before you as to whether the equine species suffers from it. The affected horse unfortunately cannot describe his sensations, and we are therefore left in doubt as to whether they are the same as in man; but, judging from appearances, we might conclude they are at least somewhat similar.

The first case to which my attention was directed, occurred so long ago as 1865, when stationed with one of my old regiments—the 3rd Hussars—at Aldershot. The horse—a dark brown gelding. rather old, if I remember aright—was off-side wheeler in the regimental drag, and had been bought at an auction in London. The officer who usually drove the drag asked me to examine the horse's left ear, as he said that when on the road the animal began to shake his head and incline it to the left, as if a fly had got into the ear and could not be dislodged. On several occasions the drag had to be stopped as the horse staggered towards the left side and could not go on. On one occasion, when urged to proceed, the horse became greatly excited and fell, and it was some time before he could be sufficiently recovered to bring him home. A careful scrutiny of the external ear, inside and out, could discover nothing to account for the symptoms; the teeth were examined, as well as the jugular veins, but they were normal. Another bridle was worn, and the horse's place in the coach changed, but all was of no avail, and as the animal gave so much trouble, he was sold. The shaking of the head and vertigo were ascribed to Megrims.

The next case presented itself in a horse of my own, when with the Royal Engineers at Chatham, 1876. The animal was an Irish hunter, seven years old, which I drove in double harness, as the hunting season was over when I bought him. He ran on the off side, and was a showy and steady horse at work. Soon after I began driving him, I found he was seized in the same way, when on the road, as the other horse had been.

When going along quietly, he would suddenly give a start, become excited, shake his head rapidly and convulsively towards the left side, carrying it much as a horse does when a twitch is severely applied to one of its ears, or as if something very irritating had entered the ear. At the same time, he pressed against the pole on the near side with such force as to drive the other horse off the road into a hedge, and then fell. The groom was quickly at his head, and we released him from harness. He did not appear unconscious, but stupefied, and his eyes moved as if he were much startled. In a few seconds he got up, but was so much depressed and agitated that we did not put him again to the carriage for some time. He would not drink water, which was procured from an inn not far off; and bathing his head did not appear to benefit him much. Next day I made a most particular examination of him, but could detect nothing whatever to cause the peculiar and alarming symptoms. The condition was not what is commonly known as Megrims, neither was it Epilepsy, several well-marked cases of which I had previously seen. The horse was otherwise in excellent health and condition.

He was put under treatment, as it was imagined his digestive apparatus might be at fault, and his allowance of oats was reduced. But this had no effect, as the next time he was driven he exhibited the same symptoms; but he was timeously pulled up, and the groom got to his head to support him; and though he had pushed the near side horse across the road, yet he was prevented from falling. This occurred several times at intervals of a few days.

Whether he was liable to these attacks in the stable, I could not learn, as the groom did not observe them there; and as I did not use him in the saddle, I am in doubt as to whether he would show the peculiar symptoms when ridden. At any rate, the condition were so alarming, and even dangerous, that I was obliged to dispose of him, as I could not afford to destroy him and make an autopsy, much as I desired to do so.

Under the heading of "Irritation in Horse's Ear," a correspondent to *The Field* of May 2d of the present year, writes: "Can any of your readers tell me the cause and the best treatment for the above? The horse is seven years old, and appears perfectly healthy; but about six months ago he first showed signs of irritation, by shaking his head and putting his near ear down. The last month the irritation has increased, and in harness now he will stop, shake his head, and twitch his ear severely. It more frequently appears when he is warmed up, or when meeting a cold wind. When riding it affects him just the same, but when I see it coming on I rub gently behind the ear, and in a minute or so it passes off. I have tried washing the ear with carbolic acid, and have also applied lead liniment. There is nothing to be seen, the ear being perfectly clean. A local veterinary surgeon has examined him, but cannot detect the cause.'' The writer subscribes himself "Puzzled.''

From the symptoms I have given of Menière's disease in man, and those I have detailed as offered by the two horses which came under my own personal observation, it will be seen that there is some reason for assuming that animals may be affected with Labyrinthine Vertigo, though what its immediate or remote causes or termination may be in them I cannot say. These certainly were not cases of ordinary vertigo. The sudden excitement as if startled by a loud noise or sharp pain; the persistent and increasing shaking of one ear, and inclination of the head to the same side; the tendency to turn round or push to that side; the stupefied and alarmed look, and the falling on that side if not quickly pulled up and supported, are indications that go, as far as is possible in dumb animals, to support the opinion I have ventured to express with regard to the existence of Menière's disease in other creatures than man.

Doubtless many members of the veterinary profession have witnessed such cases and been struck by their peculiarity, but I can find no mention of any attempt to assimilate them to, or compare them with labyrinthine disorder in the human species. Disease of, or derangement in, the organ of hearing in horses, must be exceedingly rare, though they are frequent in dogs and cats; and, as has been mentioned, acariasis of the external ear in these animals occasions symptoms not unlike those described. But in the horses I have alluded to, there was nothing whatever discovered to account for the attacks to which they were liable. Whatever the cause may have been, it was not always an operation, as the fits only came on at uncertain periods and, so far as could be ascertained, during work.

The function of the semicircular canals of the middle ear is doubtless closely connected with movement, or the consciousness or co-ordination of movement, as Flourens long ago demonstrated; for some of them causes loss of equilibrium and disturbance of locomotion; and if this section is made only in one ear, then the disturbance in equilibrium and movement occurs on that side. In the cases I have described, if the cause was really located in these canals, it may have been variation of pressure on the fluid they contain, due to some disturbance in the blood circulation or modification in the temperature or amount of air in the Eustachian tubes.

However this may be, certain it is that we have here a form of vertigo that has not been, to my knowledge, described; and whether it is altogether neurotic, and due to some morbid condition of the cerebellum, or it is the consequence of some derangement in the auditory apparatus, is a matter for investigation. I cannot think that such cases are very rare, and it is possible for some one to have the opportunity of making a careful examination of a living horse so affected, and to have the advantage of a skilfully-carried-out autopsy upon it, so that we may learn the true pathology of this condition.

With regard to treatment, I am sorry to say I cannot give any information; for until we can ascertain the cause of the malady, any remedial measures adopted must be more or less of an empirical nature. Those applied to man cannot be utilized for the horse to the same extent. It may be mentioned, however, that with man large doses of quinine have appeared to give good results. But until we understand the ætiology of the malady, we must remain in the dark as to the proper means to be prescribed for combating it.

